# Facilitating shared decision-making in oncology

**DOI:** 10.3389/fpsyg.2023.1216165

**Published:** 2023-10-10

**Authors:** Alberto Sobrero, Valentino Martelli, Alessandro Pastorino

**Affiliations:** Medical Oncology Unit 1, Ospedale Policlinico San Martino IRCCS, Genova, Italy

**Keywords:** shared decision-making, oncology, judgment, psycho oncology, perspectives

## Introduction

There is a wide consensus that the best way to practice medicine is evidence-based medicine that results from the merging of three domains: relevant scientific data, the doctor's clinical judgment, and the patient's preferences and values. These concepts were pioneered by Sackett almost 30 years ago (Sackett et al., 1996). It is surprising how little the elaboration of clinical judgment has been investigated in recent years (Charles et al., 2004; Hoffmann et al., 2014). In addition, despite the wide endorsement of shared decision-making by clinicians, patients, and their advocates (Stiggelbout et al., 2015; Elwyn et al., 2017), its translation into practice remains limited (Légaré and Witteman, 2013). This commentary addresses these issues and tries to explain why practicing full shared decision-making is so difficult in oncology.

## The challenge of shared decision-making in oncology

Aside from the intrinsic difficulty of the message to be conveyed and the huge skills needed to convey these messages in oncology (Weeks et al., 2012), the complexity of the clinical decision-making process is the major challenge.

In his latest book “Noise: A Flaw in Human Judgment,” Nobel Prize winner Daniel Kahneman demonstrates that whenever a decision must be taken by humans, including medical decisions, an unacceptably high level of variability is present (Kahneman et al., 2021). The biological mechanisms of processing information by the human brain are still largely unknown (Tongtong et al., 2022); however, they are based on the integration of concepts from psychology, behavioral sciences, anthropology, and neurobiology.

According to Kahneman, humans process, analyze, and react to facts, data, and information via two demonstrated mechanisms called systems 1 and 2 (Kahneman and Levitt, 2011). System 1 is automatic, subcortical (amygdala), and effortless, but subjected to bias and errors. In addition, this system tends to simplify things through heuristics that are cognitive shortcuts. For example, “I recently saw my sister dying from pancreatic cancer despite treatments, now that I have breast cancer I do not want to be treated, because the treatments are useless.” System 2 is much more reliable and cortical, but slow. It is typical of any fully rational decision.

We speculate that these two systems present the elaborated information to the two determinants of judgment that in humans are coupled: intelligence (the capacity to rationally solve problems) and conscience (the capacity to feel emotions and sentiments).

Reason and feelings produce judgments that are heavily influenced by a third factor that Kahneman called “narrating self,” an internal function of our mind based on memories, personal values, and preferences (Kahneman and Levitt, 2011). The narrating self is our personal power to “manipulate/re-shape/distort” the information being processed so that our judgment of data and facts fits our personal representation of reality. This is why the interpretation and reaction to the same facts, data, or information are hugely different from doctor to doctor and from patient to patient (“noise” in medicine).

Given this complexity, it is not surprising that the same data explained to patient A produce evaluations that may be completely different from those of patient B. For example, when 150 patients with stage III colon cancer who received adjuvant chemotherapy were asked what percentage cure rate would make them accept the same treatment again, about one-third would accept to be treated again for a 1% absolute reduction in risk recurrence, and about two-third believed that only a 5% reduction would justify treatment (Love et al., 2007).

This process of data interpretation by patients indicates why shared decision-making must be the outcome of evidence-based medicine. Based on decades of research (Henselmans et al., 2018; Josfeld et al., 2021; Marieke, 2022), the concept of shared decision-making is that physicians and patients collaborate in the entire process, with a continuous exchange of information. On one hand, the physician presents scientific data to the patient in the most simplest and understandable manner. On the other hand, as the patient progressively comprehends the clinical situation, he communicates his own values and preferences to the doctor.^1^

The following are the four steps that can be recognized in the shared decision-making process:

1. The doctor elaborates his own judgment for “that specific patient” on the pros and cons of the options indicated by guidelines.
2. The doctor informs and engages the patient in the most unbiased way, considering the wide range of alternatives (Elwyn et al., 2017).
3. The patient, with his personal intellectual and emotional tools, elaborates his own judgment and discusses that with the doctor (Elwyn et al., 2017).
4. The doctor and the patient come to a shared decision that derives from the two distinct processes of data interpretation and personal beliefs, thereby realizing full patient empowerment.

## Conclusion

All these steps account for the complexity that renders fully shared decision-making so difficult on top of the hard content of the information to be conveyed in oncology. Nevertheless, this task is not prohibitive and can be facilitated by a wider knowledge of these mechanisms among clinical oncologists.

## Author contributions

All authors listed have made a substantial, direct, and intellectual contribution to the work and approved it for publication.

## Footnotes

^1^ https://www.cancer.org/cancer/managing-cancer/making-treatment-decisions/making-decisions.htm
